# Targeting the Immunomodulatory CD73/Adenosine System to Improve the Therapeutic Gain of Radiotherapy

**DOI:** 10.3389/fimmu.2019.00698

**Published:** 2019-04-05

**Authors:** Simone de Leve, Florian Wirsdörfer, Verena Jendrossek

**Affiliations:** Institute of Cell Biology (Cancer Research), University Hospital Essen, University of Duisburg-Essen, Essen, Germany

**Keywords:** CD73, adenosine, radiotherapy, therapeutic window, normal tissue toxicity, T_reg_, macrophages, tumor microenvironment

## Abstract

Extracellular adenosine is a potent endogenous immunosuppressive mediator critical to the maintenance of homeostasis in various normal tissues including the lung. Adenosine is either released from stressed or injured cells or generated from extracellular adenine nucleotides by the concerted action of the ectoenzymes ectoapyrase (CD39) and 5′ ectonucleotidase (CD73) that catabolize ATP to adenosine. An acute CD73-dependent increase of adenosine in normal tissues mostly exerts tissue protective functions whereas chronically increased adenosine-levels in tissues exposed to DNA damaging chemotherapy or radiotherapy promote pathologic remodeling processes and fibrosis for example in the skin and the lung. Importantly, cancer cells also express CD73 and high CD73 expression in the tumor tissue has been linked to poor overall survival and recurrence free survival in patients suffering from breast and ovarian cancer. CD73 and adenosine support growth-promoting neovascularization, metastasis, and survival in cancer cells. In addition, adenosine can promote tumor intrinsic or therapy-induced immune escape by various mechanisms that dampen the immune system. Consequently, modulating CD73 or cancer-derived adenosine in the tumor microenvironment emerges as an attractive novel therapeutic strategy to limit tumor progression, improve antitumor immune responses, avoid therapy-induced immune deviation, and potentially limit normal tissue toxicity. However, the role of CD73/adenosine signaling in the tumor and normal tissue responses to radiotherapy and its use as therapeutic target to improve the outcome of radiotherapy approaches is less understood. The present review will highlight the dual role of CD73 and adenosine in tumor and tissue responses to radiotherapy with a special focus to the lung. It will also discuss the potential benefits and risks of pharmacologic modulation of the CD73/adenosine system to increase the therapeutic gain of radiotherapy or combined radioimmunotherapy in cancer treatment.

## Introduction

Radiotherapy is a mainstay in the treatment of cancer patients. About 60% of all cancer patients receive radiotherapy during the course of their disease alone or in multimodal combinations of surgery, radiotherapy, and chemotherapy, with beneficial effects of these highly effective treatments on long-term survival and tumor cure (1–5). Moreover, much progress has been made with technical improvements in treatment planning that increase accuracy of dose delivery, as well as by the development of particle therapy approaches (6). Nevertheless, cure rates still need to be improved for prevalent cancer types with high loco-regional failure-rates or a high risk for invasive growth or metastatic spread. For example, patients suffering from locally advanced non-small cell lung cancer (NSCLC) are typically treated with fractionated radiotherapy to the thoracic region, or concurrent platinum-based radiochemotherapy (RCT) yielding local control rates of 40–66% with doses of 60–66 Gray (Gy) (7–9). But loco-regional failures upon definitive RCT or disease progression by distant metastases are common and it is thought that improving local control rates will directly improve survival rates (9, 10).

Herein biological factors such as the high intrinsic tumor cell radio resistance, a pronounced tumor heterogeneity, diversity in radiation responses, and a resistance-promoting microenvironment reduce the efficacy of radiotherapy and thus contribute to failures. Otherwise, the high radio sensitivity of the normal lung tissue limits the application of curative radiation doses to the thoracic region and therapy intensification efforts of RCT (9, 11). Technical advances in image guidance and modern radiation techniques have significantly increased the safety profile of thoracic radiotherapy (12–14); but radiation-induced lung disease (RILD) still represents a serious normal tissue complication associated with radio(chemo)therapy of thoracic neoplasms or total body irradiation in conditioning regimens for hematopoietic stem cell transplantation (15–17). Moreover, toxicity rates can increase or new toxicities can be observed when using molecularly targeted drugs in combination with radiotherapy (18–21). Thus, there is a high need for further innovations in radiotherapy practice that improve the tumor response without increasing toxicity.

The progress in cancer immunotherapy and the discovery that radiotherapy activates T-cell-mediated antitumor immune responses under certain conditions, particularly when combined with established immune checkpoint blockade, expedited interest, and research in exploiting a potential benefit of combining radiotherapy with immunotherapy in pre-clinical and clinical cancer research (22–30). However, there are still major challenges in defining optimal dosing and treatment schedules and understanding the dual face of radiation-induced immune changes with potential impact on immune-related adverse effects. Moreover, only a fraction of patients responds to the treatment with immune checkpoint blockade alone or in combination with radiotherapy as tumors may not be immunogenic, dispose of efficient strategies to escape from tumor immune surveillance, or responses may not be durable (31–34).

In this context, accumulation of extracellular adenosine through activation of 5′ectonucleotidase (CD73) and subsequent signaling through adenosine receptors is a common mechanism how tumors escape from tumor immune surveillance. This makes CD73/adenosine signaling an attractive target in immuno-oncology and the related studies and underlying principles are well covered in various reviews (35–39).

But the role of CD73/adenosine signaling in the response of tumors and normal tissues to radiotherapy and its potential impact on the outcome of radiotherapy and combined radioimmunotherapy are less well described. Herein it is important to consider that the effects of CD73/adenosine activation on the immune system and reconstitution of tissue homeostasis might well differ among tissues of different origins as well as between acute and chronic activation stages. Therefore, we will first introduce the contribution of radiotherapy-induced changes in the innate and adaptive immune cell compartments to acute and chronic tumor and normal tissue responses and point to beneficial and adverse roles to the outcome of radiotherapy. We will then summarize current knowledge about the role of CD73 and adenosine in tumor and normal tissue responses to radiotherapy, and highlight the potential of targeting CD73/adenosine for improving the therapeutic gain of radio (immuno)therapy in thorax-associated tumors with high risk of adverse late effects in the highly radiosensitive normal lung tissue.

## Paradigm Change: Radiation Activates Local and Systemic Immune Effects

The broad use of radiotherapy as standard treatment option in the therapy of solid human tumors is based on its ability to damage cellular macromolecules, particularly the DNA, thereby effectively inducing growth arrest and cell death locally in irradiated tumor cells. But bystander effects such as the transmission of lethal signals between cells via gap junctions or the production of diffusible cytotoxic mediators can also contribute to local antineoplastic action of radiotherapy. However, despite reported transient immunosuppressive effects by local induction of immune cell death (40) and or immune impairment (41, 42), multiple reports highlight the ability of radiotherapy to induce systemic effects that involve activation of the innate and adaptive immune systems (22, 23, 43, 44).

In the context of tumor therapy, exposure to ionizing radiation can modulate immunosuppressive barriers in the tumor microenvironment, trigger the recruitment of immune effector cells to the local tumor, render tumors accessible to infiltration of immune effector cells by modulating restrictive tumor vessels, and even elicit tumor-specific immune responses leading to the regression of tumor lesions locally and at tumor sites outside the radiation field (abscopal effects) (22, 45–50). Elegant pre-clinical investigations helped to reveal the importance of T-cell responses to the local and abscopal antitumor effects in response to radio(immuno)therapy and to uncover the underlying mechanisms (47, 51–57).

Since abscopal effects seem to be rare in the clinical situation (49, 58–61), current clinical trials focus on combining radiotherapy with different immunotherapies (30, 47, 48, 62–64). Notably, there is hope from first clinical studies that blockade of the programmed cell death 1/programmed death-ligand 1 (PD-1/PD-L1) immune checkpoint might improve progression-free survival in lung patients with an acceptable safety profile, when given after radiotherapy or platinum-based RCT (65, 66). But further studies are needed to explore the efficacy and the safety profile of combined therapy of cancer patients suffering from thorax-associated neoplasms with radiotherapy and immunotherapies, to define biomarkers for patient selection and potential compensatory immune-tolerance mechanisms in malignant tumors (27, 67), and to define optimal treatment schedules.

It appears that the local induction of damage to highly radiosensitive resident cells in the lung with subsequent activation of non-targeted immune effector mechanisms might also contribute to the adverse effects of ionizing radiation in normal tissues such as the development of pneumonitis and pulmonary fibrosis (68–74). Similar to other models of sterile inflammation radiation-induced damage to resident normal lung tissue cells triggers a multifaceted damage-signaling cascade including a multifactorial secretory program in order to stimulate repair and recovery (74). However, radiation induces chronic changes in irradiated tissues that presumably result from a persistent damage signaling. These chronic environmental changes impact not only the phenotype of resident cells but also the recruitment and polarization of immune cells infiltrating the previously irradiated lung tissue, thereby disturbing the balance between inflammatory and repair processes and promoting chronic fibrosis progression (73).

## Dual Face of Radiation-Induced Immune Changes: Balance Between Immunoactivating and Immunosuppressive Effects

As outlined above, exposure to ionizing radiation has the capacity to induce immune responses in normal and tumor tissues. These changes involve a complex interplay between cells of the irradiated malignant or healthy normal tissues and cells of the innate and adaptive immune systems. But, depending on the type (tumor vs. normal) and origin of the irradiated tissue, the temporal appearance (acute vs. chronic), and the basal immune status of the tissue before exposure to ionizing radiation (pro- vs. anti-inflammatory), the response of the immune system can either adopt immunostimulatory or immunosuppressive effects and have either a positive impact (anti-tumor; normal tissue protection) or a negative impact (pro-tumor; normal tissue toxicity) on treatment outcome.

In the following paragraphs we will highlight the dual roles of the immune system in the response of tumor and normal tissues after irradiation that are mostly derived from pre-clinical studies.

### Tumor Tissue

Radiation-induced immune changes in the tumor involve the direct activation of innate and adaptive immune responses influencing tumor growth; but radiation-induced immune responses also include indirect responses such as radiation-induced changes in the tumor vasculature or tumor microenvironment that impact the recruitment and activation state of cells from the innate and adaptive immune system [for a review see (64, 75–79)].

Tumor irradiation induces damage and death of cancer cells resulting in the surface exposure of immunogenic molecules as well as the release of damage associated molecular patterns (DAMPs) such as ATP or High-Mobility-Group-Protein B1 (HMGB1), and potentially tumor antigens, to activate innate and adaptive immune responses (80). Nuclear release and cytoplasmic sensing of altered nuclear acids via Toll-like receptor (TLR)9 or cyclic GMP-AMP Synthase/Stimulator of Interferon Genes (cGAS/STING) is intimately connected to the secretion of cytokines that support innate and adaptive antitumor immunity. Priming of tumor-specific T cell responses requires uptake of tumor antigens by antigen presenting cells e.g., dendritic cells. Furthermore, priming of tumor-specific T cells depends on sensing of cancer-cell derived cytoplasmic DNA. e.g., by the cGAS/STING pathway that is connected to the activation of the interferon (IFN) I response to support antitumor immunity. The initiated migration and antigen presentation of dendritic cells then triggers the activation of B and T cells in secondary lymphoid organs. Activated T and B cells subsequently exert anti-tumor effects by several mechanisms like CD8^+^ T cell mediated cytotoxicity, antibody-dependent cell-mediated cytotoxicity, and antibody-induced complement-mediated lysis. These processes have been excellently described in more detail elsewhere (27, 43, 47, 57, 75, 80–82).

Thus, the direct induction of anti-tumor immunity in response to ionizing radiation requires a complex interplay between the innate and adaptive immune system and the tumor microenvironment. Moreover, the recruitment and activation of dendritic cells in irradiated tumors that are required for the priming of tumor-specific T cell responses largely depends on the dose and fractionation of radiation in a tumor-dependent manner. Finally, tumor cells dispose of multiple mechanisms to evade this response so that the direct induction of anti-tumor immunity by radiotherapy is a rare event (41, 57, 83).

Besides these beneficial radiation-induced anti-tumor immune responses, local irradiation can also induce subacute or chronic immune changes that mostly exert tumor-promoting effects. Pro-inflammatory cytokines released in tissues as a damage response after radiotherapy as well as the humoral immune response from activated B cells can activate cells of the innate immunity, such as granulocytes, macrophages, and mast cells (84, 85). These cells release molecules that modulate gene expression programs in favor of pro-survival signaling and cell cycle progression in neoplastic cells thereby supporting malignant tissue expansion (86, 87). Moreover, cells from the innate immunity have the capacity to induce repair, regeneration, and tissue remodeling. By releasing various mediators these cells influence and initiate fibroblast activation, angiogenesis and matrix metabolism thereby indirectly fostering tumor growth (84, 88–90).

Finally, the tumor itself responds to radiation-induced stress or damage through a panel of phenotypic changes. By releasing several cytokines, chemokines, or growth factors as well as up-regulating specific surface receptors e.g., immunosuppressive PD-L1, cytotoxic T-lymphocyte-associated Protein 4 (CTLA-4), carcinoembryonic antigen-related cell adhesion molecule 1 (CEACAM1), and others, tumor cells become proficient in dampening immune responses and to escape the immune system (91–96). Detailed reviews from Sharma et al. as well as Wennerberg et al. recently summarized the role of these tumor cell-extrinsic factors for primary and adaptive resistance so that these mechanisms will not be further addressed here (34, 41).

### Normal Tissue

Despite technological improvements ionizing radiation still directly hits to some extent tumor-surrounding healthy tissue during treatment, leading to local damage, stress, or cell death in normal resident cells. Moreover, the damage response initiated in malignant tissues and healthy tissues residing in the radiation field not only contributes to the local effects of radiotherapy but can also exert strong systemic effects promoting normal tissue complications (22, 97–99).

Radiation-induced immune changes in normal tissues also include acute and chronic immune effects that will be discussed below. While the effects of radiation-induced normal tissue inflammation are well described (100–106), the contribution of the complex immune mechanisms that support chronic, pathological changes e.g., fibrosis, are less investigated and still not yet completely understood.

Similar to the situation in tumors, radiation-induced acute damage and cell death in normal tissues also results in the release of DAMPs as well as pro-inflammatory cytokines and chemokines which have the capacity to modulate immune responses (105, 107). These “danger signals” trigger the activation and influx of innate and adaptive immune cells at the site of damage resulting in normal tissue inflammation. An excessive inflammation during the acute phase after radiation as a result of an overwhelming secretion of pro-inflammatory cytokines and the release of reactive oxygen species (ROS) supports normal tissue toxicity and severe side effects in treated patients (108).

In addition DAMPs can activate tissue regeneration in normal tissues as well as in tumor tissues. It is known that the extracellular DAMPs HMGB1 and ATP can activate and recruit cells, thus stimulating tissue repair (109). Of these, innate immune cells invade into the damaged tissue to clear dead cells and cellular debris (110). Moreover, stem cells and tissue-associated cells, e.g., fibroblasts, keratinocytes, endothelial cells, and vascular smooth muscle cells, are stimulated by these DAMPs to support angiogenesis and tissue regeneration (111–117). In addition, several DAMPs (e.g., HMGB1, S100A4, uric acid) can also enhance the expression of immunosuppressive mediators like interleukin (IL)-10 and indoleamine 2,3-dioxygenase (IDO) in stem cells, thereby inhibiting lymphocyte responses and contributing to tumor promotion (118). Excellent detailed reviews about the role of DAMPs in mediating regenerative pathways can be found elsewhere (109, 119).

Radiation-induced damage to normal tissues furthermore triggers chronic environmental changes e.g., hypoxia and senescence, that are reminiscent of the changes observed in the tumor microenvironment. These changes support chronic inflammation and repair processes, promote alternative polarization of recruited immune cells, pathologic immune cell interactions and excessive tissue remodeling, and thereby trigger not only the development of tissue scaring and fibrosis but also the development of secondary tumors (120, 121). For more detailed reviews about the acute and chronic events during radiation-induced normal tissue toxicity please refer to Wirsdorfer and Jendrossek (73), McKelvey et al. (79), Schaue et al. (105), Stone et al. (122), Barnett et al. (123), Kim et al. (124), and Ruhle and Huber (125).

The dual face of radiotherapy-induced immune changes in normal tissues can be nicely demonstrated in murine models of RILD. The acute phase of pneumonitis and the chronic event of fibrosis dramatically reveal how complex the radiation-induced alterations of the tissue micromilieu and the immune system impact disease pathogenesis (73).

Own studies in the murine C57BL/6 model of RILD revealed that whole thorax irradiation (WTI) with 15 Gy triggered an acute early immune suppression characterized by a pronounced reduction in the number of lymphocytes and myeloid cells that was followed by an influx of CD3^+^ T cell lymphocytes into the lung tissue during the pneumonitic phase Interestingly, WTI also enhanced numbers of regulatory T cells (T_reg_) in the lung tissue of irradiated animals both, during the early inflammatory state as well as at the time of fibrosis development. Of note, radiation-induced pulmonary fibrosis was more severe in recombination-activating gene 2 (RAG2)-deficient mice that lack mature T- and B-lymphocytes suggesting that lymphocytes may have beneficial effects (126). Instead, depletion of CD4^+^ T cells during the pneumonitic phase decreased radiation-induced lung fibrosis in rats pointing to a contribution of CD4^+^ T cells to disease pathogenesis (68). These data suggest a causal link between the recruitment or local expansion of specific T-lymphocyte populations and the course of RILD that are also observed in other fibrosis models (127). But further work is required to define the beneficial and adverse effects of recruited and induced T cell subsets during the course of RILD (128).

Thoracic irradiation induces not only changes in the T cell compartment but also in the myeloid compartment and the lung environment. For example, others and we detected a significant reduction in the levels of total pulmonary macrophages and an almost complete eradication of alveolar macrophages early after irradiation as well as time-dependent changes in the macrophage phenotype with increased expression of markers for alternative macrophage activation [e.g., macrophage mannose receptor and Arginase-1] particularly during the fibrotic phase (102, 126, 129–132). Further, macrophages accumulated in organized clusters and expressed pro-fibrotic mediators such as alpha smooth muscle actin (α-SMA) and transforming growth factor beta (TGF-β) at ≥25 weeks post-irradiation (131). Importantly, a recent report confirmed the formation of organized clusters of CD163^+^ macrophages also in lung tissue of irradiated patients. Intriguingly, pharmacologic inhibition of colony stimulating factor-1 (CSF-1) inhibited macrophage influx and attenuated RT-induced lung fibrosis in mice supporting a pathologic relevance of macrophages in RT-induced lung fibrosis (132). Similarly, pharmacological treatment with as connective tissue growth factor (CTGF) antibody before or after 20 Gy thoracic irradiation reduced acute and chronic radiation toxicity in mice and abrogated M2-like macrophage infiltration (133). The combined inhibition of TGF-β and Platelet-Derived Growth Factor (PDGF) blockade in a pre-clinical murine model attenuated radiation-induced pneumonitis and lung fibrosis and was accompanied by reduced osteopontin expression and leukocyte infiltration (134). Instead strategies using anti- Vascular Endothelial Growth Factor (VEGF) to target the tumor vasculature in combination with radiotherapy turned out to be highly toxic to normal lung tissue in pre-clinical murine models (21).

## The Immunomodulatory CD73/Adenosine System as Therapeutic Target for Improving Radiotherapy Outcome

Various observations from pre-clinical and clinical studies summarized in the former paragraphs suggest that targeting tumor-induced or radiation-induced immune deviation may offer novel and attractive opportunities for improving the outcome of radiotherapy by modulating the tumor radiation response, radiation-induced adverse late effects, or both. But the complexity of the tumor-induced and radiation-induced changes in the microenvironment as well as the time- and tissue-dependent “dual face” of radiotherapy-induced immune changes highlight the importance to identify strategies suited to balance adverse pro-inflammatory and immunosuppressive effects of radiotherapy and outweigh the beneficial effects of radioimmunotherapy with optimal tumor control and normal tissue protection. In this context, the purinergic CD73/adenosine system recently moved into the focus of research as it is an important endogenous regulator of the innate and adaptive immune systems with a documented role in tumor immune escape but also in adverse late effects of radiotherapy (36, 38, 131, 135–138).

We therefore hypothesized that the purinergic system might offer novel opportunities to interfere with normal tissue and tumor responses to radiotherapy and radiation-induced immune deviation. Extracellular ATP is a danger signal released by dying and damaged cells, and belongs to the earlier mentioned DAMPs, that function as immunostimulatory pro-inflammatory signals (139). In contrast, extracellular adenosine mostly exerts anti-inflammatory, immunosuppressive or regulatory functions and is a critical mediator for the maintenance of tissue homeostasis in various tissues including the lung and to avoid overwhelming inflammation for example in response to infection (140–143). But balancing pro-inflammatory ATP and anti-inflammatory adenosine might also to be crucial for maintaining or re-establishing immune homeostasis and to orchestrate tissue inflammation and repair under conditions of damage-induced sterile inflammation (73, 144).

### CD73 and Adenosine Have Physiological Roles in Maintaining and Restoring Tissue Homeostasis

The purinergic signaling pathway is an evolutionary conserved mechanism that regulates immune homeostasis by the conversion of extracellular ATP to extracellular adenosine by using the sequential degradation via the ectoenzymes ectoapyrase (CD39, ectonucleoside triphosphate diphosphohydrolase 1) and CD73. Adenosine is either released from stressed or injured cells, or generated from extracellular adenine nucleotides by the concerted action of CD39 and CD73. While CD39 catalyzes the breakdown of ATP and ADP to AMP, CD73 converts AMP to adenosine. But the action of CD39 in degrading ATP can alternatively be executed by ectonucleotide pyrophosphatase (ENPP1, phosphodiesterase 1) (145).

CD39 and CD73 are expressed on the surface of specific lymphocyte subpopulations such as T_reg_ and regulatory B cells (B_reg_) and endothelial cells and are important to their regulatory functions (143, 146–149). But CD73 is also expressed on stromal cells, mesenchymal stem cells (MSCs), and tumor-associated stem cells (150–153). Pre-clinical studies demonstrated that CD73 on stromal cells and tumor cells participates in the suppression of immune-mediated responses (152) as well as in homing and stemness of cancer stem cells (151, 154, 155). Furthermore, CD73 on MSCs promoted their immunosuppressive function and MSC were even able to upregulate CD73 expression on T cells (150). Inhibition of CD73 in a pre-clinical model of pancreatic neuroendocrine tumors led to reduced tumor growth and metastatic potential of cancer stem cells (151). Thus, stem cell-mediated immunosuppressive or regenerative processes might help cancer cells to escape natural anti-tumor immune responses, anti-cancer immunotherapies, or both. Table 1 shows detailed information on the expression of CD73 on multiple cell types in various tissues and their reported prognostic findings. Adenosine suppresses inflammatory functions of cells from innate and adaptive immune system and triggers expansion or differentiation of myeloid-derived suppressor cells (MDSC), M2-like macrophages as well as T_reg_ and B_reg_ and thereby participates in the creation of regulatory environments (144, 146, 149, 180–184). In addition, CD39/CD73-dependent generation of adenosine may also affect other processes in T-cell biology such as naive T-cell homeostasis, memory cell survival, and potentially T cell differentiation (168).

**Table 1:** CD73 expression on various cell types and tissues and its prognostic finding.

**Cell type**	**Tissue or cell type**	**Origin**	**Reported prognostic finding**	**References**
Monocyte	Peripheral blood post-infarcted myocardium	Human Swine	Mesenchymal Stem Cells Induce Expression of CD73 in Human Monocytes *in vitro* and in a Swine Model of myocardial Infarction *in vivo*. Positive ADO loop leads to attenuation of inflammation and promotes the regeneration of the damaged myocardial tissue	(156)
	Monocytes in the inflamed joint	Murine	CD73 expression is associated with the suppression of inflammation in rheumatoid arthritis	(157)
Neutrophil	Neutrophils in the inflamed joint	Murine	CD73 expression is associated with the suppression of inflammation in rheumatoid arthritis	(157)
Dendritic cell	Skin	Murine	Production of Extracellular Adenosine by CD73^+^ Dendritic Cells Is Crucial for Induction of T cell Anergy and Tolerance in Contact Hypersensitivity Reactions	(158)
MDSC	MDSCs generated from mouse hematopoietic progenitor cells (*in vitro*)	Murine	Generation of ADO by CD73 may promote MDSC expansion and facilitate their immunosuppressive activity	(159)
	Peripheral Blood from advanced melanoma patients	Human	High baseline levels of CD73 on MDSCs negatively correlate with Overall Survival and Progression Free Survival	(160)
Macrophage	Alveolar macrophages	Murine	CD73 expression in the lung tissue contributes to radiation-induced lung fibrosis	(138)
	Peritoneal macrophages	Murine	CD73 regulates anti-inflammatory signaling between apoptotic cells and endotoxin-conditioned tissue macrophages and is required to limit neutrophil influx in a peritonitis model	(161)
NK cells	Peripheral blood 2–5% CD73^+^ NK cells	Human	ADO induces T cell suppression	(162–164)
	Upregulation of CD73 upon exposure to MSC (*in vitro*)	Human	CD73^+^ NK cells have the potential to regulate NK cell activation in an autocrine or paracrine manner	(165)
B cells	Subpopulations of murine memory B cells, germinal center B cells	Murine	ADO signaling is prominent in the mature germinal center and required for establishment of the long-lived plasma cell compartment	(166)
	Peripheral blood and tonsil	Human	Dependence of Immunoglobulin Class Switch Recombination in B Cells on Vesicular Release of ATP and CD73 Ectonucleotidase Activity Common variable immunodeficiency (CVID) patients with impaired class-switched antibody responses are selectively deficient in CD73	(167)
	Colon B cells	Murine	B cell CD73/CD39/adenosine mediates immunosuppression in DSS-induced colitis	(149)
T cells	Th1, Th2, Th17, Treg in normal, and tumor tissues	Murine human	CD73 may favor cell homeostasis, memory survival, and differentiation	(168–170)
		Human	CD73^+^ T cells infiltrate into breast and ovarian tumor tissue	(165)
		Human murine	ADO induces immunosuppression	(146, 171–174)
		Murine	CD73 expression on extracellular vesicles derived from Treg contributes to their regulatory function	(175)
		Murine	CD73 expression in the lung tissue contributes to radiation-induced lung fibrosis	(138)
Fibroblasts	Cancer-associated fibroblasts in High-grade serous ovarian cancer (HGSC)	Human	High CD73 expression on CAFs is associated with worse prognosis	(176)
	Cancer-associated fibroblasts in bladder cancer	Human	High CD73 expression on CAFs is associated with worse prognosis	(177)
Epithelial cells	Retinal pigment epithelial cells	Murine	CD73 expression is associated with the suppression of conventional CD4 cell proliferation	(178)
	Renal epithelial cells	Murine	CD73 expression on proximal tubular epithelial cells Is critical in renal ischemia-reperfusion injury protection	(179)
Endothelial cells	Bladder cancer	Human	High CD73 expression is associated with better survival in non-muscle-invasive BC (NMIBC) and muscle-invasive BC (MIBC) tumors	(177)
Mesenchymal stem cells (MSC)	Experimental autoimmune uveitis (EAU)	Murine	Inhibition of T-cell proliferation	(150)
Stem cells (Hematopoitic stem cells; cancer stem cells)		Murine human	CD73/Ado induce stemness, homing	(151, 154)

Extracellular adenosine can either be removed by enzymatic inactivation or cellular uptake or exert its actions through receptor binding. Adenosine deaminase (ADA) is responsible for the conversion of adenosine to inosine, a process that can happen either extracellularly or intracellularly (144). Adenosine may also be transported into its target cells via four different adenosine transporters, the so-called equilibrative nucleoside transporters (ENT) 1-4. Instead, adenosine exerts its actions by binding to one of four different G-protein-coupled adenosine receptors (ADORA1, ADORA2A, ADORA2B, and ADORA3) that are widely expressed on immune cells and resident tissue cells (185). ADORA1 and ADORA2A are high-affinity receptors responding to low concentrations of extracellular adenosine, while ADORA2B and ADORA3 are low affinity receptors and are mainly activated if the extracellular adenosine concentration rises above physiological levels (186). The adenosine receptors have various biological functions aimed at maintaining or restoring tissue homeostasis by triggering context-dependent pro- or anti-inflammatory effects (187–189).

### Role of CD73 and Adenosine in Radiation-Induced Adverse Late Effects in the Lung

There is evidence from pre-clinical studies in models of injury-induced sterile inflammation that an acute CD73-dependent increase in adenosine mostly exerts tissue protective functions (142, 181, 190, 191). Herein, the role of purinergic signaling to self-terminate TLR-responses in macrophages might contribute to the observed effects (187, 192).

In contrast, chronically increased adenosine-levels induced for example by genetic deficiency of the adenosine-degrading enzyme ADA or chronic treatment with the chemotherapeutic drug Bleomycin (BLM) can promote pathologic remodeling processes in various tissues leading to fibrosis development (136, 193–200). The pathologic effects of BLM-induced chronic adenosine-accumulation in the lung have been attributed to alternatively activated myeloid cells (201, 202).

So far the role of purinergic signaling for radiation-induced adverse late effects in the lung has only been addressed in own investigations (131, 138) while others investigated its role the skin (136). Our work demonstrated a pathologic role of chronically increased CD73/adenosine signaling in irradiated lungs of C57BL/6 mice, presumably by promoting or amplifying profibrotic signaling cascades. Pathologic signaling involved a time-dependent increase in the expression and activity of the CD73 in the lung tissue that could be confined to resident CD45^−^ cells as well as CD45^+^ immune cells (CD4^+^ T cells including T_reg_, alveolar macrophages) and was associated with a progressive increase in adenosine levels in the bronchioalveolar lavage fluid C57BL/6 mice with a knockout of CD73 (CD73^−/−^) failed to accumulate high levels of adenosine in response to WTI resulting in decreased levels of fibrosis-associated proteins and mediators, reduced recruitment/formation of T_reg_, and attenuated pulmonary fibrosis with absence of clusters with alternatively activated macrophages. A similar protective effect was obtained by treatment of irradiated C57BL/6 mice either with pegylated ADA (PEG-ADA) to catabolize adenosine, or with the CD73 monoclonal antibody (mAb) TY/23 as of week 16 post-irradiation (131, 138).

**Figure 1:**
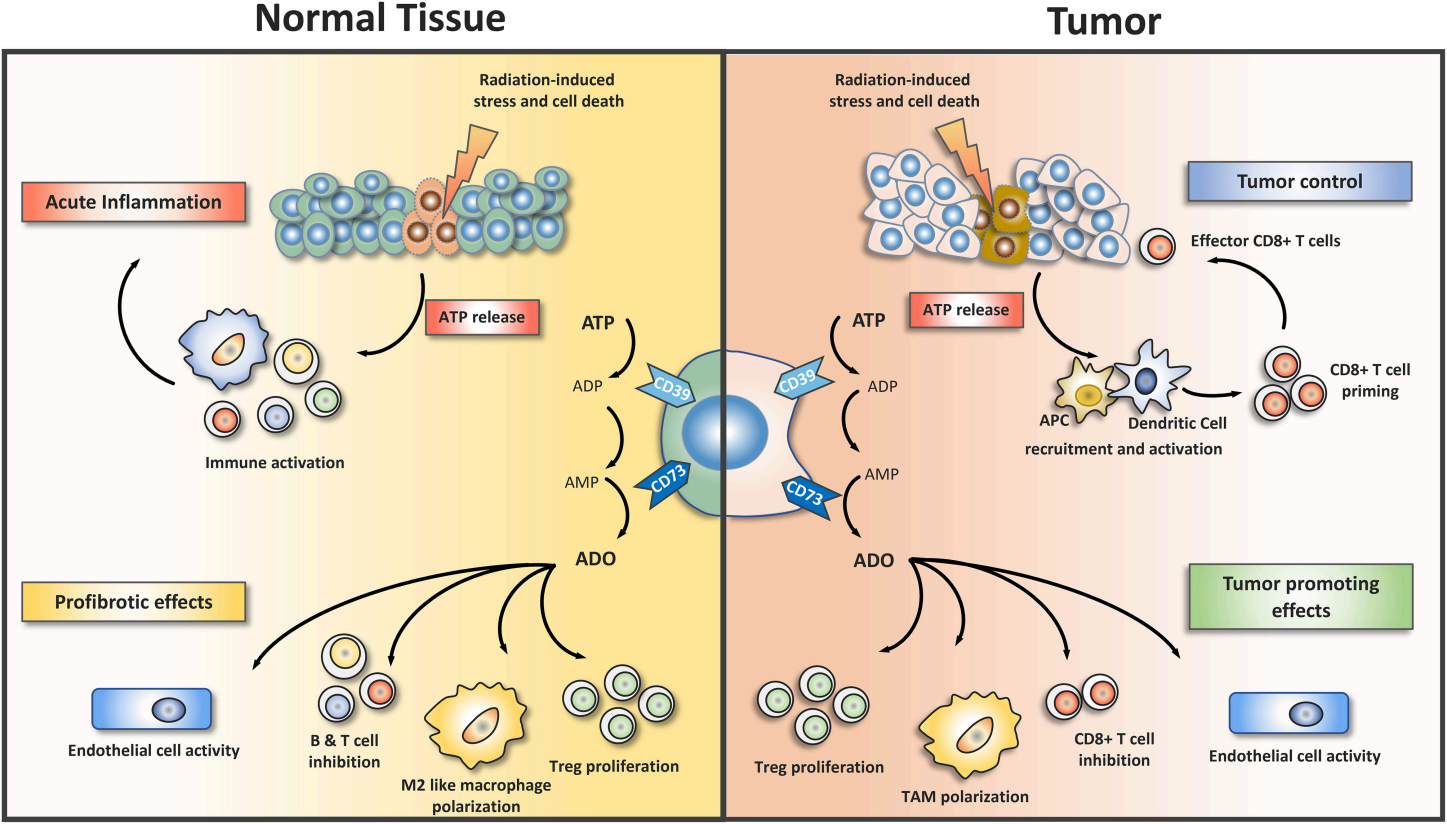
Purinergic signaling shapes the microenvironment in irradiated normal and tumor tissues. Exposure of normal tissues to ionizing radiation induces damage to tissue resident cells, e.g., endothelial cells and epithelial lung cells, as well as in resident immune cells. Equally exposure of tumor tissue results in radiation-induced damage to tumor cells and stromal cells. The resulting cell damage initiates stress responses and/or cell death with subsequent release of damage associated molecular patterns (DAMP). Release of ATP from dying cells is one component of radiation-induced tissue damage. Extracellular ATP acts as a potent inflammatory mediator that promotes inflammation and subsequent further damage to normal tissues. In tumor tissues extracellular ATP is an important mediator of anti-tumor CD8^+^ T cell responses as it participates in activation of dendritic antigen presenting cells (APC). To avoid excessive inflammation in normal tissues pro-inflammatory ATP is rapidly removed from the extracellular room by a two-step enzymatic conversion into adenosine, involving CD39 (or alternatively ectonucleotide pyrophosphatase) and CD73. Extracellular adenosine is an important endogenous regulator of inflammatory and repair processes as well as vascular functions. Adenosine exerts its pleiotropic actions in a tissue- and context-dependent manner through 4 different adenosine receptors that are expressed on various resident cells and immune cells (not shown). The immunosuppressive actions of adenosine involve the polarization of recruited immune cells toward regulatory or alternatively activated phenotypes, e.g., regulatory T cells (T_reg_), or M2-like macrophages. Moreover, adenosine mediates the inhibitory action of T_reg_ and other regulatory cell types on proliferation and activation of cytotoxic T cells. By regulating endothelial cell activity CD73 and adenosine impact not only endothelial cell proliferation/angiogenesis but also vascular barrier function and the transmigration of leukocytes into damaged tissues. The expression of CD39 and CD73 thus balances the levels of pro-inflammatory ATP and immunosuppressive adenosine in normal tissues and tumors. The chronic activation of adenosine-driven processes observed in irradiated normal tissues promotes pathologic tissue remodeling and fibrosis development. Tumors coopt the CD73/adenosine system as a mechanism for promoting tumor growth and progression, angiogenesis, and immune escape. ADO, adenosine; CD39, ectonucleoside triphosphate diphosphohydrolase 1; CD73, 5′ ectonucleotidase; TAM, tumor associated macrophages.

Taken together, the progressive up-regulation of CD73/adenosine signaling in the irradiated lung environment promotes the accumulation of immunosuppressive cell types of the innate and adaptive immune system, e.g., T_reg_ and M2-like macrophages and supports a pro-fibrotic cross-talk between damaged resident cells and infiltrating immune cells. Thereby, CD73/adenosine signaling helps to amplify radiation-induced lung fibrosis as a late normal tissue complication (Figure 1). In support of our findings, adenosine also promoted radiation-induced skin fibrosis; but here the pro-fibrotic effects had mainly been attributed to T-cell infiltrates and signaling via ADORA2A, without a role for alternatively activated macrophages (136).

Although radiation-induced intestinal injury models exist, the role of CD73/adenosine has only been studied so far in acute inflammatory disease models where CD73/adenosine executes tissue protective functions (203–208). Moreover, while CD73/adenosine had protective effects in acute renal disease models, chronic kidney injury in patients and murine studies was again linked to up-regulation of CD73 and ADORA2B (179, 198, 209, 210).

In summary, these studies point to disease-promoting effects of chronic CD73/adenosine signaling with tissue-specific and damage-specific mediators and immune changes.

### Role of CD73 and Adenosine in the Control of Tumor Growth and Response to Therapy

Analyses of patient biopsies have shown that immune cell infiltrates in human tumors exhibit pronounced differences in cell types and numbers, not only intratumorally but also between patients and different tumor entities (211, 212). Interestingly, distribution and type of infiltrating immune cells turned out to have prognostic relevance; for example, the presence of infiltrating T cells was mostly linked to a favorable clinical outcome (213–218). Further pre-clinical and clinical studies showing that tumors can be strongly or poorly immunogenic supported these findings (31, 219). Moreover, the degree of immunogenicity positively correlated with reduced tumor growth and increased survival of tumor-bearing mice in response to immunotherapy indicating that the immune status can be seen as a predictive factor for therapy outcome (220).

High numbers of tumor-infiltrating cytotoxic lymphocytes were also predictive for the response of head and neck cancer patients to treatments involving radiotherapy whereas relapse after chemoradiotherapy and early recurrence correlated to infiltration with myeloid cells (217, 221–224). Local or systemic increases in T_reg_, high numbers of tumor associated macrophages, or recruitment of CD11b^+^ myeloid cells have also been associated with poor tumor response to radiotherapy and tumor relapse in pre-clinical models (88, 225). As a proof of concept for the synergistic interaction of radiotherapy and immunotherapy it has been shown that the combination with cancer vaccines, immune checkpoint blockade or inhibition of CD11b^+^ cell recruitment can improve the outcome of radiotherapy (52, 62, 89, 226–228).

Of note, tumors coopt the activities of the purinergic CD39/CD73/adenosine system to shape the immune landscape in the tumor microenvironment at multiple levels (Figure 1): For example, tumor cells and tumor-associated T_reg_ use CD73-dependent adenosine generation to dampen intratumoral immune responses, particularly in hypoxic tumors (229, 230). The re-direction of the immune response involved suppression of T cell effector functions through CD73-dependent production of extracellular adenosine by CD39^+^/CD73^+^ T_reg_ and signaling via stimulation of the ADORA2A on effector T cells (229). Adenosine and ADORA2A thus participate in shaping an immunosuppressive tumor microenvironment by negatively regulating CD8^+^ T cells (231–233). An adenosine-dependent suppression of immunosurveillance via IFN-γ, NK cells, and CD8^+^ T cells had also been demonstrated in other pre-clinical models (35, 162). Finally, the creation of an immunosuppressive tumor microenvironment involved the expansion of immunosuppressive myeloid cells, e.g., myeloid-derived suppressor cells, M2-like macrophages, and potentially N2-like neutrophils (234–236). More details about the various effects of CD73 and adenosine on cells from the innate and adaptive immune systems in the tumor microenvironment and the involved ADOR receptors can be found in the following reviews: (137, 143).

In addition, the CD73/adenosine system also supports tumor growth-promoting neovascularization, tumor metastasis, and chemotherapy resistance though part of these actions could also be attributed to the CD73/adenosine-induced modulation of immune cell types in the tumor microenvironment (36, 143, 229, 237–244).

For example, CD73^−/−^ mice were strongly resistant to growth of subcutaneous MC38-ova colon and EG7 lymphoma tumors as well as carcinogen-induced or *de novo* growth of endogenous prostate tumors in transgenic TRAMP mice (162, 245, 246). These interesting observations pointed to a role of CD73^+^ host cells in tumor growth. However, CD73^−/−^ mice were less resistant to growth of AT-3 mammary and B16F10 melanoma tumors revealing that the effect of host CD73 on the growth of experimental tumors also depends on the tumor type (245, 246). Of note, treatment with an anti-CD73 mAb reduced the growth of experimental 4T1.2 and E0771 breast tumors in wild-type mice, but not in severe combined immunodeficient (SCID) mice, suggesting a role of the adaptive immune system (245, 246). Anti-CD73 treatment also inhibited growth of carcinogen-induced fibrosarcoma tumors and of transgenic prostate tumors in transgenic TRAMP mice (162). The authors could further attribute the efficient tumor rejection to the action of CD8^+^ T cells whereas CD4^+^ T cells and NK cells were not involved (162, 246). These data highlight immunosuppressive CD73^+^ T_reg_ as an important component of the tumor growth-promoting effects of CD73 and adenosine (162, 246).

Interestingly, CD73^−/−^ mice also developed less lung metastases after intravenous injection of B16F10 or TRAMP-C1 cells (162, 246) suggesting that host CD73 also supports metastasis. In line with these observations treatment with an anti-CD73 mAb (TY/23) strongly reduced the lung metastases after injection of 4T1.2 or TRAMP-C1 tumor cells (162, 245). However, the suppression of metastasis formation was observed in both, immunocompetent and in SCID mice, and turned out to be independent of CD8^+^ T cells and NK cells (162, 245). Thereby the authors revealed a role of CD73^+^ non-hematopoietic host cells in metastasis formation, potentially endothelial cells, they could further link the pro-metastatic effect to signaling of tumor-derived extracellular adenosine via ADORA2B activation, at least in the 4T1.2 model (245, 246).

In further studies, tumor-derived adenosine attracted myeloid cells and promoted their differentiation into adenosine-generating tumor-associated macrophages (TAM) to amplify adenosine-dependent tumor-immune escape (247). In support of these findings, *in vitro* exposure to adenosine promoted alternative activation of macrophages and enhanced the immunosuppressive responses of macrophages to danger signals, particularly if stimulated in the presence of TLR ligands (141, 187). Interestingly, tumor-derived CD73-dependent adenosine promoted growth, neovascularization, and metastasis of subcutaneous B16F10 melanoma tumors and this was linked to infiltration and polarization of macrophages: genetic or pharmacologic inhibition of CD73 on the B16F10 melanoma cells significantly reduced the number of tumor-infiltrating macrophages recruited to subcutaneous B16F10 melanoma tumors on CD73^−/−^ mice when compared to untreated B16F10 wildtype tumors on CD73^−/−^ mice. Cytokine measurements in CD73^+^ B16F10 wildtype tumor lysates grown on CD73^−/−^ mice revealed a down-regulation of pro-inflammatory cytokines [Granulocyte-macrophage colony-stimulating factor (GM-CSF) and IFN-γ] and enhanced expression of anti-inflammatory/pro-angiogenic cytokines (IL-4, IL-10, IL-13, M-CSF) (248). Although the number of infiltrating macrophages did not change in CD73^+^ B16F10 WT tumors on CD73^−/−^ mice, less MMR^+^ macrophages were found inside the tumor. Only a pharmacological CD73 inhibition or knockdown of CD73 in the tumor host reduced the amount of infiltrating macrophages (248, 249). The results indicate a role for CD73 in activation and polarization of macrophages that promote tumor progression. Furthermore, it was shown, that the recruitment and activation of tumor-infiltrating macrophages was dependent on ADORA1, ADORA2A, and ADORA3 (250).

Taken together, CD73-dependent adenosine from host cells and tumor cells participates in the support of tumor growth amongst others by promoting tumor immune escape whereas loss of CD73/adenosine signaling enhances tumor immunity. As nicely summarized in a recent review from Allard et al. CD73/adenosine has become an attractive therapeutic target in (immuno)-oncology (38). Several early-phase clinical trials currently evaluate the therapeutic potential of CD73/adenosine inhibitors to inhibit tumor growth and increase tumor immunity. Besides the direct inhibition of CD73 the identification of the respective ADOR involved in promoting tumor immune escape will offer additional opportunities for therapeutic intervention (38, 251, 252).

Intriguingly, co-inhibition of adenosine signaling via CD73 and ADORA2A achieved better anti-tumor immune responses compared to single treatments, at least in pre-clinical models of breast and colon cancer (253). These effects were associated with improved immune cell infiltration, DC-priming and CD8^+^ T cell expansion. In line with these findings, Young et al. also observed increased tumor growth delay in CD73/ADORA2A double knockout mice (254). Furthermore, investigations with the human monoclonal anti-CD73 antibody MEDI9447 that is currently in Phase I clinical trials, showed high efficacy in inhibiting CD73 *in vitro* and potent inhibition of pre-clinical syngeneic tumor models *in vivo* as well as additive activity in combination with immune checkpoint inhibitors. Interestingly, MEDI9447 efficiently modulated the tumor microenvironment with significant alterations in the number of both, CD8^+^ effector T cells and activated macrophages (255).

The immunosuppressive actions of CD73 and adenosine in the microenvironment of established tumors also attract major attention as an interesting target for combined treatment approaches, particularly with immunotherapy. In this context, inhibition of CD73 enhanced efficacy of immunotherapy with α-PD-1 or α-CTLA4 in pre-clinical models (251, 256). The synergistic effect of the combined treatment involved improved T cell effector function as well as reduced CD73 expression on tumor-infiltrating lymphocytes and was dependent on interferon gamma (IFN-γ) and perforin (253, 256). Therapeutic inhibition of ADORA2A was also able to modulate expression of T cell co-inhibitory receptors and to improve effector function for enhanced efficacy of immune checkpoint blockade and adoptive cell therapy in murine cancer models (251, 252).

Since the focus of this review is to highlight the therapeutic potential of CD73 and adenosine inhibition to improve the therapeutic gain in radiotherapy we will not discuss such approaches in more detail here. For further information please refer to reviews discussing the therapeutic potential of the purinergic pathway in immunotherapy in more detail (38, 251, 252).

Instead the therapeutic potential of combining radiotherapy or radioimmunotherapy with CD73/adenosine-inhibition in cancer has been highlighted as an attractive approach but sound data are missing so far (41, 137). Pre-clinical studies are now underway to test such approaches including investigations in our own laboratory (257).

Several pre-clinical studies addressing the role of CD39 in cancer revealed that genetic deficiency of CD39 in mice promotes resistance to metastasis of melanoma and colorectal cancer models (258). Similarly, inhibition of angiogenesis in a CD39-deficient background resulted in reduced growth and pulmonary metastasis of LLC and B16F10 tumors (259). Expression of CD39 was important for angiogenesis and the suppression of NK cell-mediated antitumor activity (260, 261). In line with these findings, overexpression of CD39 enhanced the metastatic potential in pre-clinical models whereas the pharmacological inhibition of CD39 reduced metastasis and enhanced antitumor immunity (261). Of note, clinical data also support a correlation of high CD39 expression with poor prognosis indicating that CD39 might be another promising target for cancer therapy (262–264). But CD39 is much less investigated as a cancer target compared to CD73 underlining the need for further pre-clinical studies.

Taken together various pre-clinical studies highlight the potential of CD39/CD73/adenosine-signaling as promising therapeutic target in immuno-oncology. So far, the observed effects have been associated in multiple studies with activation of T-cell dependent tumor immunity. However, it is important to consider further immunoregulatory actions of CD73 and adenosine in the tumor microenvironment, particularly their influence on the biology of myeloid cells and macrophages, respectively.

### Targeting CD73 in Lung Cancer

Only limited data are available so far of the role of CD73 and adenosine in lung cancer. Herein, CD73 was found to be expressed in tumor tissue from NSCLC patients on tumor cells, tumor-promoting mesenchymal stromal cells and myeloid-derived suppressor cells, respectively (265–267). Tumor-derived TGF-β stimulated CD39 and CD73 expression in CD11b^+^CD33^+^ MDSC in tumor tissues and peripheral blood of NSCLC patients, thereby inhibiting activity of T cells and NK cells and protecting tumor cells from the cytotoxic effect of chemotherapy (267).

**Figure 2:**
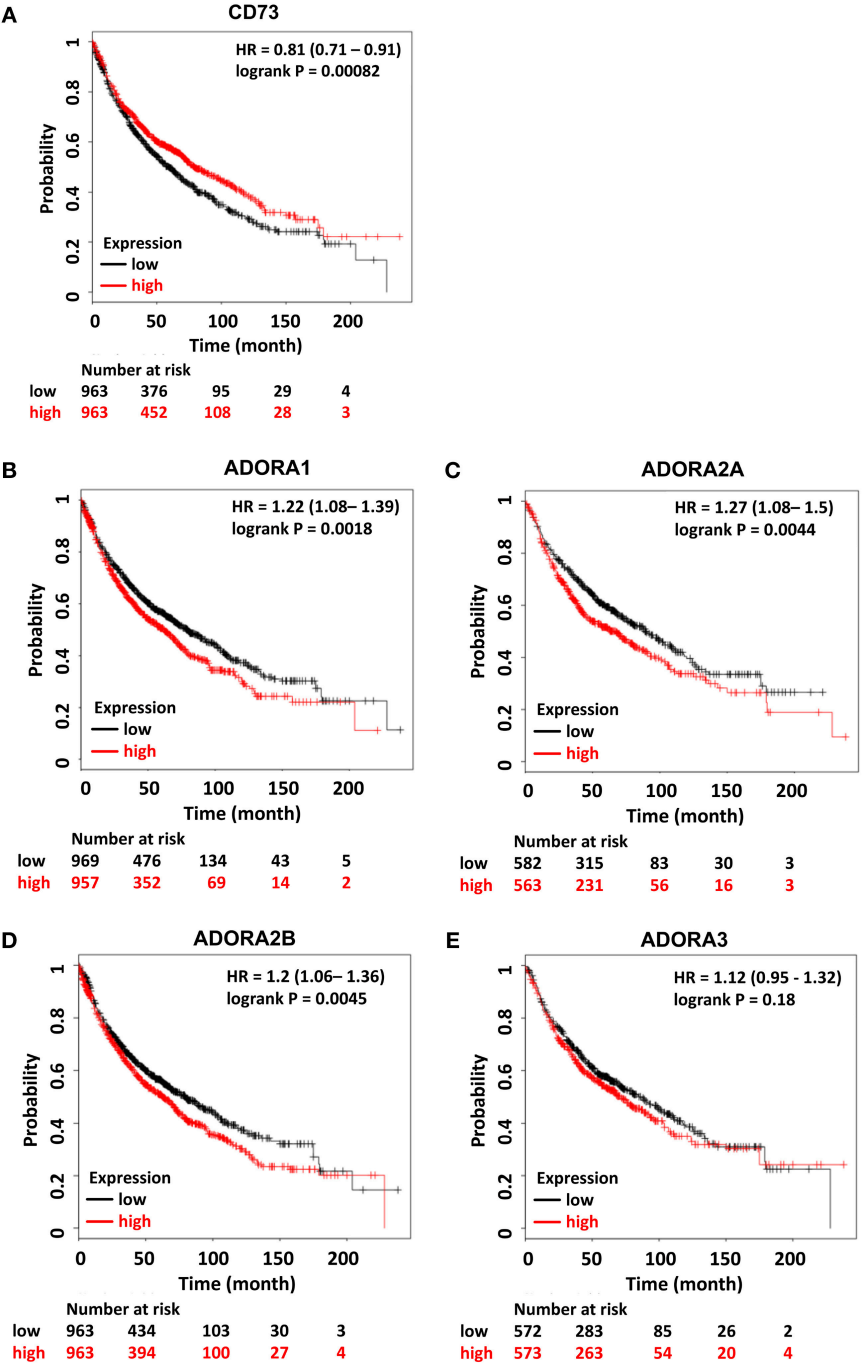
Prognostic relevance of components of the CD73/adenosine signaling system in lung cancer. Kaplan–Meier survival curves relative to **(A)** NT5E, **(B)** ADORA1, **(C)** ADORA2A, **(D)** ADORA2B, and **(E)** ADORA3 expression from publically available datasets for lung cancer. Data were analyzed using the KM-plotter tool (270). Red and black lines indicate patients with higher and lower gene expression, respectively. The total number of patients in the two categories are shown below the graph. Hazard ratios (HR) and *p*-values (log rank p) are shown inside the graph. Patient data is not restricted and includes all datasets.

Moreover, the prognostic value of high CD73 expression for the survival in lung cancer patients remains controversial: Although one study reported a correlation of high CD73 gene expression and improved overall survival of NSCLC patients (268) another study identified high CD73 protein expression as an independent prognostic marker for poor overall survival and shorter recurrence free survival in NSCLC (269). Interestingly, in the same study, high ADORA2A gene expression was an independent predictor of favorable prognosis for overall survival (269). Own *in silico* analyses of publicly available datasets for gene expression of CD73 in lung cancer confirmed the positive correlation between high CD73 gene expression and better overall survival of NSCLC patients. Of note, if radiotherapy-treated patients were excluded from the analysis the correlation to an improved overall survival was abrogated. In addition, the *in silico* analyses revealed poorer overall survival in lung cancer patients with high gene expression of ADORA1, ADORA2A, and ADORA2B (Figure 2). Again, the results about the prognostic value of ADORA2A using immunohistochemical data revealed opposite results (269, 270). The discrepancy in the above findings may be due to the use of gene expression analyses vs. immunohistochemical data as CD73 expression in tumor samples turned out to be highly heterogenous (269). We speculate that the heterogeneity in CD73 protein expression in distinct tumor areas might be linked to heterogeneous tumor oxygenation and make the acquisition of representative gene expression data challenging. So far, CD39 inhibitors are not yet involved in clinical trials for cancer patients but such studies are underway (259).

Taken together, adenosine released in an inflammatory milieu or generated by the CD39/CD73 axis will impact the immune landscape of lung tumors presumably by limiting T cell immunity and promoting immunosuppressive and tumor-promoting lymphoid and myeloid immune cell phenotypes (Figure 1). We thus speculate that modulating CD73/adenosine signaling in the lung tumor microenvironment is an attractive strategy to limit tumor progression, improve antitumor immune responses, and avoid escape from therapy in combination with radiotherapy and potentially radioimmunotherapy. On the other hand, the pathologic role of the radiation-induced increase in CD73/adenosine signaling in promoting chronic inflammation and fibrosis in the normal lung tissue strongly suggest that pharmacologic inhibition of CD73/adenosine offers the opportunity for widening the therapeutic window by reducing radiation-induced lung toxicity, particularly in CD73-rich thoracic tumors with a high risk for CD73-dependent normal tissue toxicity.

Targeting the CD73/adenosine pathway or the involved receptors may thus provide a clear therapeutic gain in the treatment of lung cancer and other CD73/adenosine-rich thorax-associated neoplasms: we expect that inhibition of CD73/adenosine signaling will limit lung toxicity during thoracic irradiation without protecting the tumor or even reinstall anti-tumor immunity when applied during therapeutic irradiation of adenosine-rich tumors with high radioresistance such as NSCLC (138). However, a tight regulation of pro- and anti-inflammatory actions of resident and immune cells is necessary to protect the lung from inflammation-induced loss in its vital function (271–273). For example, immunosuppressive T_reg_ are also to be part of a protective response limiting inflammation-induced collateral normal tissue damage after radiotherapy (44, 274). Therefore, pharmacologic strategies targeting the CD73/adenosine pathway in combination with radiotherapy or combined radioimmunotherapy will require careful validation of potential normal tissue complications. Such complications might include excessive inflammation or autoimmunity by abrogating protective signals mediated by various ADORAs, particularly during acute disease stages. Moreover, the dual effects of acute and chronic CD73 activation as well as spatiotemporal heterogeneity of CD73 and ADOR expression in normal and tumor tissues need to be considered when designing combination treatments for therapeutic intervention.

## Current Research and Future Perspectives

So far work from our group identified CD73/adenosine signaling as a novel mechanism promoting RILD through local and systemic actions. Consequences of pathologic CD73/adenosine signaling involved amongst others the accumulation and/or alternative activation of macrophages in organized clusters, their expression of pro-fibrotic mediators, or both. We speculate that the radiation-induced increase in CD73/adenosine is necessary to amplify pro-fibrotic signaling in the irradiated lung environment by fueling the multifaceted cross-talk between damaged resident cells, local and infiltrating immune cells, immunosuppressive T_reg_ and other pro-fibrotic mediators such as hyaluronic acid and TGF-β.

Though immunomodulatory effects of adenosine had been linked to CD73/adenosine-induced adverse effects in other injury models (136, 202) the tissue specific effector and target cells of CD73/adenosine-signaling in response to genotoxic treatment (BLM, radiotherapy) are still controversial and need to be further investigated (73, 200). In this context radiation-induced normal tissue toxicity had also been linked to endothelial cell damage and dysfunction as well as endothelial cell loss as long-term complication (275, 276). As a direct consequence of impaired vascular function, WTI increased numbers of total CD45^+^ leukocytes, particularly profibrotic CD11b^+^ myeloid cells and Ly6C^+^ inflammatory monocytes, in lungs of irradiated mice. However, on the long term, persistence of an activated pro-coagulant endothelial cell type, thickening of the basement membrane, endothelial loss, and collapse of microvessels will contribute to the creation of a hypoxic, pro-inflammatory disease-promoting environment. We assume that the pathologic environment involves a hypoxia-induced up-regulation of CD73 and pathology-associated ADOR on resident cells and immune cells. It is tempting to speculate that therapeutic inhibition of CD73 might also impact adverse late effects in the lung by reducing radiation-induced vascular impairment, but this remains to be determined. Interestingly, further work demonstrates that locally irradiated MSC play a role in the pathogenesis of radiotherapy-induced pulmonary fibrosis by acquiring a pro-fibrotic myofibroblast-like phenotype that promotes extracellular matrix deposition, tissue remodeling, and the development of pulmonary fibrosis upon WTI (276). Since CD73 is expressed on endothelial cells and on MSC of healthy lungs (153) future studies should explore whether the expression of CD73 on the surface of endothelial cells or resident MSC impacts the development of RILD. The same holds true for the expression of CD39 and CD73 on cancer exosomes, which have also been shown to suppress T cells through adenosine production (239).

Adenosine released in an inflammatory milieu or generated by the CD39/CD73 axis impacts the tumor microenvironment and limits tumor immunity at multiple levels. Thus, modulating cancer-derived adenosine in the tumor microenvironment emerges as an attractive strategy to limit tumor progression and improve antitumor immune responses and our own studies suggest that this might be possible without excessively increasing late normal tissue complications (36, 187, 242, 243, 277). Fortunately, multiple approaches for pharmacologic modulation of adenosine levels exist or are being developed and multiple clinical studies have been initiated to evaluate the use of novel inhibitors of CD73 or ADORA2A signaling in cancer therapy alone and in combination with immune checkpoint blockade (38, 39, 143, 188). These studies will give insight into efficacy, compatibility, and potential side effects.

Herein, major attention in oncology has so far been attributed to adenosine signaling via ADORA2A as it is known to effectively dampen immune responses in tumors and normal tissues. However, it has to be taken into account that depending on the tissue of origin and the molecular and immune signature of the tumor, other ADOR may be more important. Moreover, the role of purinergic signaling in the radiation response of malignant tumors and the potential of CD73 or ADOR inhibitors to enhance the efficacy of RT alone and in combination with immunotherapy is still largely unknown. Finally, no reliable biomarkers for the prediction or diagnosis for the individual risk of RILD upon treatment are available to date. Thus, further studies are needed that correlate the gene and protein expression of CD73 and the ADORAs to the outcome after radio(chemo)therapy or immunotherapy. Moreover, as mentioned before, the receptors differ in their affinity for adenosine and extracellular adenosine levels will vary depending on the tissue, the treatment modality and intensity in a spatiotemporal manner. It would therefore be highly beneficial to perform an immunoscore of tissues from pre-clinical studies and test association of high or low expression of CD73 and the ADORAs with the presence of immunosuppressive lymphoid and myeloid cell subsets, and potentially tissue hypoxia. Such knowledge could later be translated into patient samples. Here, it was an intriguing observation that a high expression of CD73 in normal tissues was indicative for a poor infiltration of prostate tumors with CD8^+^ T cells whereas high CD73 expression in the tumor stroma was indicative for a longer recurrence-free survival (278). This highlights that CD73 expression in both, normal and tumor tissue should be evaluated.

## Final Remarks

Nowadays it is increasingly recognized that strategies for a biology-based optimization and individualization of radiotherapy should include not only the available knowledge about tumor promoting mutations, tumor heterogeneity, tumor cell plasticity, and unfavorable gene expression profiles indicative of the individual radiosensitivity of tumor and normal tissues, but also consider knowledge about the modulation of the radiation response by the immune system and vice-versa. Such a comprehensive view shall allow to harness the combined potential of high precision local radiotherapy, cytotoxic chemotherapy, molecularly targeted small molecule signal transduction inhibitors, and immunotherapy approaches for biologically optimized therapeutic strategies with acceptable safety profile and durable responses in the future (22, 30, 41, 279–284).

The observation that radiotherapy can help to reactivate anti-tumor immunity in immunogenic tumors or increase the potential of immunotherapy has attracted major attention to the use of radiotherapy in combination with various immunotherapies, particularly immune checkpoint blockade immunostimulatory antibodies, and cancer vaccines (24–26, 28–30, 62, 67). However, tumors have evolved effective strategies to escape from immune surveillance and therapy-induced enhancement of tumor immunity is balanced by feed-back inhibition of immune activation in residual tumors, the mobilization of tissue regeneration mechanisms with tumor promoting actions, or both (41, 88, 89, 285–287).

We believe that the identification of mediators driving both, adverse immune changes in irradiated normal tissues and tumor immune escape, will allow us to uncover attractive new therapeutic targets for improving the outcome of radiotherapy. The CD73/adenosine pathway is such a signaling system that regulates adverse immune responses in tumors and normal tissues to microenvironmental stress (e.g., tumor hypoxia) and radiotherapy. So far, CD73/adenosine is mostly considered as a metabolic immune checkpoint that supports immunosuppressive signaling of T_reg_ via ADORA2A. However, there is evidence that CD39, CD73 and adenosine are involved in further immunosuppressive and tumor-promoting signals in the tumor microenvironment beyond modulating T_reg_ function. Intriguingly, radiochemotherapy was also shown to trigger up-regulation of CD73 and CD39 in circulating immune cells of cancer patients (288). This suggests that a radiotherapy-induced systemic upregulation of CD73/adenosine signaling may additionally dampen systemic anti-tumor immune responses during standard fractionated radiotherapy.

Thus, pharmacologic inhibition of CD73/adenosine signaling is an attractive approach to increase the therapeutic ratio in the RT of thoracic tumors with high risk of adverse late effects in the highly radiosensitive normal lung tissue by (i) dampening growth and metastasis of lung tumors, (ii) enhancing the radiation-induced activation of the antitumor immune response, (iii) by restricting the immunosuppressive action of CD39/CD73 on circulating immune cells, and (iv) attenuating adverse late effects in the lung. Moreover, pharmacologic modulation of CD73, adenosine or the four adenosine receptors might offer opportunities to enhance the potential of combined radioimmunotherapy to mount efficient and durable responses with acceptable safety profile.

But the complexity of the tumor-induced and radiation-induced changes in the microenvironment and the multifaceted interactions between damaged resident cells and recruited immune cells outlined above underline the necessity of further work suited to identify strategies that achieve the required balance between pro-immunogenic and immunosuppressive effects of radiotherapy and outweigh the beneficial effects of radioimmunotherapy with optimal tumor control and normal tissue protection. Moreover, further work is required to gain a better mechanistic understanding of the tissue-, injury-, and disease stage-dependent beneficial or adverse effects of CD73/adenosine as well as the identification of involved ADORAs and effector cells for a successful restriction of lung damage during therapeutic lung irradiation by targeting CD73, adenosine or specific ADORAs (73).

Finally, it remains to be determined which approach for targeting the CD73/adenosine axis might be best suited to be used in combination with RT. Above all, the immune effects of RT also depend on physical parameters such as total dose, fractionation schemes (43, 57, 225, 289) and potentially the quality of radiation (290). Thus, attention has also to be given to the best sequence of application, as well as appropriate radiation doses and fractionation-schemes as they may largely impact the effects of radiotherapy on microvessels, immunogenic cell death, immune cell infiltration, the production of immune modulatory mediators, and the activation of CD73/adenosine signaling in both, normal and tumor tissues. Here, the major challenge will be to therapeutically redirect the immune response toward anti-tumor action and avoid tumor recurrence without enhancing collateral normal tissue damage.

## Author Contributions

All authors listed have made a substantial, direct and intellectual contribution to the work, and approved it for publication.

### Conflict of Interest Statement

The authors declare that the research was conducted in the absence of any commercial or financial relationships that could be construed as a potential conflict of interest.

## Abbreviations

ADA: adenosine deaminase
ADOR: adenosine receptor
α-SMA: alpha smooth muscle actin
BLM: Bleomycin
B_reg_: regulatory B cells
CD39, ectoapyrase: ectonucleoside triphosphate diphosphohydrolase 1
CD73: 5′ ectonucleotidase
CEACAM1: carcinoembryonic antigen-related cell adhesion molecule 1
cGAS/STING: cyclic GMP-AMP Synthase/Stimulator of Interferon Genes
CSF-1: colony-stimulating factor 1
CTGF: connective tissue growth factor
CTLA-4: cytotoxic T-lymphocyte-associated Protein 4
DAMPs: damage associated molecular patterns
ENPP1: ectonucleotidepyrophosphatase (phosphodiesterase 1)
ENT: equilibrative nucleoside transporter
Gy: Gray
HMGB1: high-mobility-group-protein B1
IDO: indoleamine 2,3-dioxygenase
IFN-γ: interferon gamma
IL: Interleukin
mAb: monoclonal antibody
MDSC: myeloid-derived suppressor cells
MMR: macrophage mannose receptor
MSC: mesenchymal stem cell
NSCLC: non-small cell lung cancer
PD-1: programmed cell death 1
PD-L1: programmed death-ligand 1
PEG-ADA: pegylated ADA
PDGF: platelet-Derived Growth Factor
RILD: radiation-induced lung disease
ROS: reactive oxygen species
RT: Radiotherapy
SCID: severe combined immunodeficiency
TAM: tumor-associated macrophages
TGF-β: transforming growth factor beta
TLR: toll-like receptor
T_reg_: regulatory T cells
VEGF: vascular Endothelial Growth Factor
WTI: whole thorax irradiation.
